# Medulloblastoma outcome is adversely associated with overexpression of EEF1D, RPL30, and RPS20 on the long arm of chromosome 8

**DOI:** 10.1186/1471-2407-6-223

**Published:** 2006-09-12

**Authors:** Massimiliano De Bortoli, Robert C Castellino, Xin-Yan Lu, Jeffrey Deyo, Lisa Marie Sturla, Adekunle M Adesina, Laszlo Perlaky, Scott L Pomeroy, Ching C Lau, Tsz-Kwong Man, Pulivarthi H Rao, John YH Kim

**Affiliations:** 1 Texas Children's Cancer Center, Department of Pediatrics, Baylor College of Medicine, Houston, TX, USA; 2 Department of Molecular and Human Genetics, Baylor College of Medicine, Houston, TX, USA; 3 St Jude Children's Research Hospital, Baton Rouge Affiliate, Baton Rouge, LA, USA; 4 Department of Radiation Oncology, Massey Cancer Center, Virginia Commonwealth University, Richmond, VA, USA; 5 Program in Neuroscience, Department of Neurology, Children's Hospital, Harvard Medical School, Boston, MA, USA; 6 Department of Pathology, Baylor College of Medicine, Houston, TX, USA

## Abstract

**Background:**

Medulloblastoma is the most common malignant brain tumor of childhood. Improvements in clinical outcome require a better understanding of the genetic alterations to identify clinically significant biological factors and to stratify patients accordingly. In the present study, we applied cytogenetic characterization to guide the identification of biologically significant genes from gene expression microarray profiles of medulloblastoma.

**Methods:**

We analyzed 71 primary medulloblastomas for chromosomal copy number aberrations (CNAs) using comparative genomic hybridization (CGH). Among 64 tumors that we previously analyzed by gene expression microarrays, 27 were included in our CGH series. We analyzed clinical outcome with respect to CNAs and microarray results. We filtered microarray data using specific CNAs to detect differentially expressed candidate genes associated with survival.

**Results:**

The most frequent lesions detected in our series involved chromosome 17; loss of 16q, 10q, or 8p; and gain of 7q or 2p. Recurrent amplifications at 2p23-p24, 2q14, 7q34, and 12p13 were also observed. Gain of 8q is associated with worse overall survival (p = 0.0141), which is not entirely attributable to MYC amplification or overexpression. By applying CGH results to gene expression analysis of medulloblastoma, we identified three 8q-mapped genes that are associated with overall survival in the larger group of 64 patients (*p *< 0.05): eukaryotic translation elongation factor 1D (*EEF1D*), ribosomal protein L30 (*RPL30*), and ribosomal protein S20 (*RPS20*).

**Conclusion:**

The complementary use of CGH and expression profiles can facilitate the identification of clinically significant candidate genes involved in medulloblastoma growth. We demonstrate that gain of 8q and expression levels of three 8q-mapped candidate genes (*EEF1D*, *RPL30*, *RPS20*) are associated with adverse outcome in medulloblastoma.

## Background

Medulloblastoma is the most common malignant brain tumor of childhood. Treatment with surgery, radiation, and chemotherapy successfully cures many patients, but survivors can suffer significant long-term toxicities affecting their neurocognitive and growth potential. Despite clinical advances, up to 30% of children with medulloblastoma experience tumor progression or recurrence, for which no curative therapy exists [1]. The lack of more effective, less toxic therapies and the inability to stratify patients biologically result from imperfect understanding of the molecular processes that underlie medulloblastoma growth [1-3].

The advent of genomic technologies has permitted a more global approach to tumor classification, diagnosis, and prognostication [4-6]. Several medulloblastoma series have been analyzed for copy number aberrations (CNAs) with comparative genomic hybridization (CGH), widely considered a standard method for genome-wide screening [7-12]. Using CGH, we have determined that gain of the long arm of chromosome 8 is associated with overall and progression-free survival of medulloblastoma patients.

Despite its limited resolution, chromosomal CGH can guide gene expression analysis, suggesting potential oncogenes or tumor suppressor genes involved in tumor growth. Our identification of 8q gain as clinically significant prompted the search for candidate genes mapped to that chromosomal region. Previous reports have identified *MYC *amplification and overexpression as clinically significant. However, we did not note such associations in our series. We investigated the possibility of other 8q-mapped candidate genes by exploiting the microarray analysis of an overlapping set of medulloblastoma specimens to complement the scale of our CGH dataset.

Of the 71 tumors we analyzed by CGH, a subset of 27 were among 64 medulloblastomas previously analyzed using gene expression microarrays, which permitted integrated analysis of both sets of data [6]. We identified three 8q-mapped genes overexpressed in tumors with 8q gain and whose expression levels were significantly associated with overall survival in all 64 patients: eukaryotic translation elongation factor 1D (*EEF1D*), ribosomal protein L30 (*RPL30*), and ribosomal protein S20 (*RPS20*). We corroborated the expression of each candidate gene in microarray analysis by quantitative real time-RTPCR (qRT-RTPCR). By analyzing expression microarray data and cytogenetic profiles, we have identified a group of clinically significant genes involved in translational regulation that display relatively small expression changes and previously eluded implication by single platform approaches.

## Methods

### Patient samples

**Figure 1:**
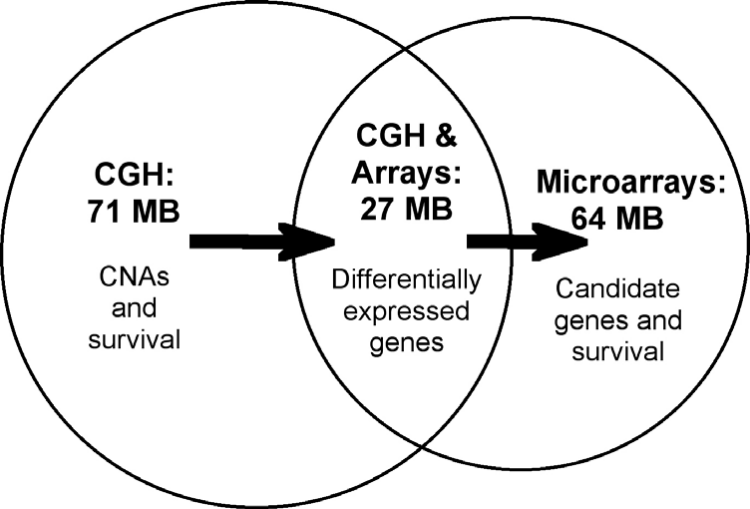
**Experimental design**. Genomic characterization of 71 medulloblastomas was performed with CGH and the most frequent CNAs were subjected to survival analysis. Twenty-seven tumors also had gene expression profile data, and were used to identify differentially expressed genes based on tumor chromosomal profiles. For survival analysis, the 64 medulloblastoma patients were stratified according to the expression values for each candidate gene.

Seventy-one medulloblastoma samples were obtained following informed consents via Institutional Review Board (IRB)-approved protocols from patients (aged 7 months – 38 years) diagnosed between 1991 and 2004 at Children's Hospital (Boston, MA) and Texas Children's Hospital (Houston, TX). Of these, 27 tumors were also previously analyzed by expression profiling using oligonucleotide microarrays [6] [Figure 1]. All specimens were obtained at the time of diagnosis prior to radiation or chemotherapy and subjected to histopathologic review according to WHO criteria [3]. Tumor samples were snap-frozen and stored in liquid nitrogen.

All patients were initially treated with maximally feasible surgical resection. Subtotal or partial resection was achieved in eleven patients. Chemotherapy for most patients (n = 50) consisted of repeated cycles of cisplatin and vincristine, with combinations of carboplatin, etoposide, cyclophosphamide and/or lomustine [13-16]. The other twenty-one patients received intensified chemotherapy cycles (vincristine, cisplatin, cyclophosphamide, and etoposide) with autologous stem cell support [17]. Patients greater than 36 months old received craniospinal irradiation 2,400 ± 360 centiGray (cGy) with a tumor dose of 5,300 ± 720 cGy. For the entire group, median follow up was 36 months (mean 39 months, range 4 – 109). All studies were performed with the approval of the Committee for Clinical Investigation and the IRB of Boston Children's Hospital, Harvard Medical School, and the IRB of Baylor College of Medicine for Texas Children's Hospital.

### Comparative Genomic Hybridization (CGH) and survival analysis

Tumor DNA was extracted using the DNeasy Tissue Protocol (Qiagen, Valencia, CA). In cases of limited tissue, DNA was extracted using TRIZOL according to the manufacturer's instructions (Invitrogen, Carlsbad, CA), or from paraffin-embedded tissues using standard methods with proteinase K digestion, phenol: chloroform extraction, and ethanol precipitation [18]. Total cellular RNA from a matching portion of frozen tissue was extracted using TRIZOL or RNeasy (Qiagen). The established medulloblastoma cell line Daoy (American Type Culture Collection, Manassas, VA) was maintained to provide control RNA, as previously described [19].

Normal metaphase spreads and chromosomal CGH were performed on 71 tumor specimens as previously described [20]. Briefly, DNA from normal female human placental controls and medulloblastoma samples were labeled by nick-translation with Texas red-5-dUTP and fluorescein (FITC)-12-dUTP, respectively (Dupont NEN, Boston, MA), and co-hybridized onto normal metaphase chromosomes. Images from at least ten to fifteen separate metaphases were captured by CCD camera (SenSys Photometrics, Tucson, AZ) mounted on a Nikon Eclipse 800 microscope. The fluorescence signal ratio (FITC:Texas red) along each chromosome was calculated with Quantitative Image Processing System (Applied Imaging, Santa Clara, CA). The average of fluorescence ratios and their 99% confidence intervals were determined for each tumor specimen. Threshold ratio values greater than 1.2 and lower than 0.8 defined tumor gains and losses, respectively. High-level amplification was defined as fluorescence ratios in excess of 2.0 at the chromosomal site.

For survival analysis of each CNA, patients were stratified into two groups based on the presence or absence of the specific cytogenetic lesion. Only those lesions detected in a minimum of five patients (7%) were analyzed. Clinical outcome measures, including overall and progression-free survival, were determined from the date of diagnosis using the method of Kaplan and Meier (SPSS v.11; SPSS, Inc, Chicago, IL). The significance of survival differences between patient groups was calculated by log-rank test. To test if 8q gain was an independent prognostic factor, we also performed multivariate analysis using Cox proportional hazard model for 8q gain with respect to three other clinical prognostic factors: extent of resection, age relative to 3 years and metastatic stage at diagnosis.

### Gene expression microarray analysis and candidate gene identification

Twenty-seven of the 71 tumors characterized by CGH were previously analyzed for their gene expression profiles on Affymetrix HuGeneFL microarrays and were among the series of 64 reported by Pomeroy *et al*. [6]. We normalized the CEL files of 64 tumors and four normal cerebellar controls. The probe set intensities used for comparisons were calculated by model-based expression index using dCHIP software (v1.3, ) [21]. Among the 44 tumors that were collected subsequently and not included in the previous report, seven were analyzed on the Affymetrix U133 Plus 2.0 microarrays and their resulting datasets were processed similarly for direct comparison with parallel qRT-RTPCR results.

Differentially expressed candidate genes mapped to 8q were subsequently analyzed for overall and progression-free survival in the larger group of 64 profiled tumors based on their expression levels (Kaplan-Meier test, SPSS). Patients were stratified into two groups based on their tumor expression level relative to that mean value. Candidate genes achieving *p *values less than 0.05 (log-rank test, SPSS) were validated using qRT-RTPCR. Fisher's exact test was used to calculate the significance of the association between gene expression levels with tumor and clinical variables.

### Quantitative Real Time-RTPCR (qRT-RTPCR)

Tumor RNA was analyzed by qRT-RTPCR performed in a Bio-Rad iQ4 Multicolor Real Time iCycler (Bio-Rad Laboratories, Hercules, CA). Total cellular RNA was reverse transcribed with M-MLV Reverse Transcriptase enzyme (Invitrogen) and oligo-(dT)12, according to the manufacturer's recommendations. PCR reactions containing cDNA, iQ Syber Green Supermix (Bio-Rad), and primers for *EEF1D, RPL30*, *RPS20, MYC*, or *MYCN *were performed in triplicate for 40 cycles (95°C 15 sec, 60°C 1 min). Amplification products were verified by melting curves, agarose gel electrophoresis, and sequencing. Copy numbers were internally normalized to *GAPDH *expression, quantitated relative to the Daoy cell line as a calibrator tissue control, and accounting for differences in primer efficiencies [22]. Results from at least three separate experiments were analyzed. Fisher's exact test was used to calculate the significance of the association between gene expression levels with tumor and clinical variables. We determined correlation coefficients (R^2^) between expression levels detected by microarray and by qRT-RTPCR using the Pearson method (relative to the Daoy cell line as a tissue control). Primer sequences are: *EEF1D*, sense 5'-CCCGCGTCCGCCGATTCCTC-3' and antisense 5'-CGCTGGCGCCGTTCTCCTG-3'; *RPL30 *sense, 5'-TGGTGGCTGCAAAGAAGAC-3' and antisense 5'-GCAGTTGTTAGCGAGAATGAC-3'; *RPS20 *sense, 5'-CACGCTCCTGCTCCTGACTC-3' and antisense 5'-GCGGCTTGTTAGGCTGATTCG-3'; *MYC *sense, 5'-TCTGGATCACCTTCTGCTGG-3' and antisense 5'-TGTTGCTGATCTGTCTCAGG-3'; *MYCN *sense, 5'-AGAGGACACCCTGAGCGATT-3' and antisense 5'-TCTTGGGACGCACAGTGATG-3';*GAPDH *sense, 5'-AAGGTGAAGGTCGGAGTCAA-3' and antisense, 5'-AATGAAGGGGTCATTGATGG-3'.

## Results

### Patient analysis and clinical data

We analyzed overall and progression-free survival for patient groups stratified by clinical variables such as age, sex, metastatic (Chang) stage, histologic subtype, and treatment regimens. Not surprisingly, metastatic disease at diagnosis displayed a trend toward worse survival [Table 1]. For other reported risk factors, only age less than three years was weakly associated with adverse clinical outcome, which could be attributed to the lack of craniospinal radiation therapy in these patients. Neither sex nor tumor histology correlated with outcome. Furthermore, different treatment centers and regimens (standard dose chemotherapy plus radiation vs. intensified chemotherapy with autologous stem cell support) were not associated with differences in survival.

### Overview of CNAs in medulloblastoma

**Figure 2:**
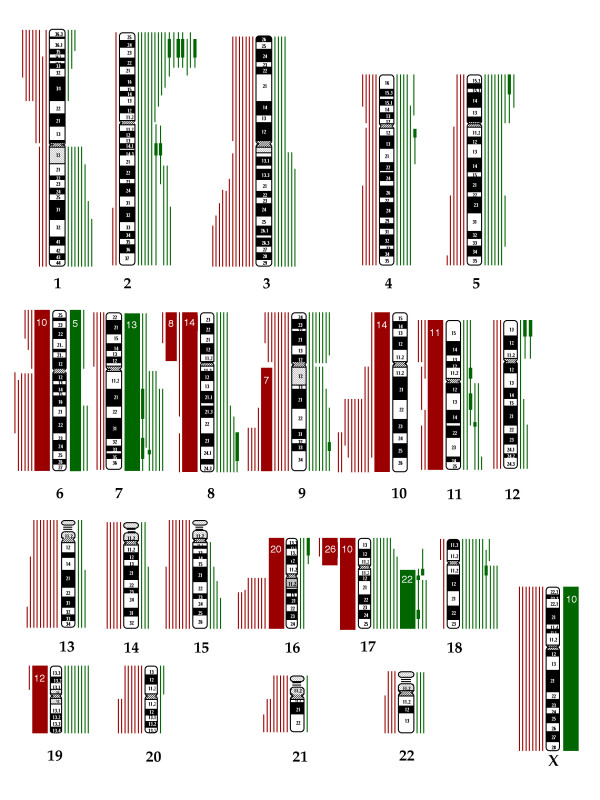
**Summary of Comparative Genomic Hybridization**. Ideogram showing genetic imbalances detected in 71 medulloblastoma by comparative genomic hybridization (CGH). Red bars on the left of the chromosomes represent losses and green bars on the right correspond to gains of chromosomal regions in tumors; amplifications are depicted as bold green bars. Multiple cases with the same CNA are grouped as thick bars, with total number of cases displayed at the top.

The 71 medulloblastomas analyzed by chromosomal CGH displayed a variety of CNAs [Figure 2; Table 2]. The most frequently observed CNAs involved chromosome 17 (i.e. loss of the short arm, 17q gain). The loss of 17p accompanied by concurrent gain of 17q, consistent with isochromosome 17q (i17q), was detected in 32% of tumors. Losses were more frequently observed than gains and predominated on chromosomes 16, 10, 8, and 11.

We also detected gain of 2p in 22% of tumors, including 5.6% with amplification at 2p23-p24, which was the most common amplification detected in our series (n = 4). Other recurrent amplifications were detected at 2q14, 7q34, and 12p13 in two specimens each and at 8q23-q24 in one tumor. Given the resolution of chromosomal CGH (approximately 10–20 Mb), the 2p23-p24 amplifications and the 8q23-q24 amplification probably include the *MYCN *(2p24.1) and *MYC *(8q24.12-q24.13) loci, respectively. Amplifications and low copy number gains at 2p23-p24 or 8q23-q24 were not associated with large cell or anaplastic histology.

### CNA-based survival analysis reveals association with gain of 8q

To evaluate the clinical significance of common CNAs, we stratified 71 medulloblastoma patients according to the presence or absence of specific cytogenetic lesions in their tumors. Specific CNAs involving 58 chromosomal regions were observed in a minimum of 7% tumors and used to stratify patients for Kaplan-Meier survival analysis [Table 2]. The most common lesions, including loss of 17p and/or gain of 17q, were not significantly associated with outcome.

**Figure 3:**
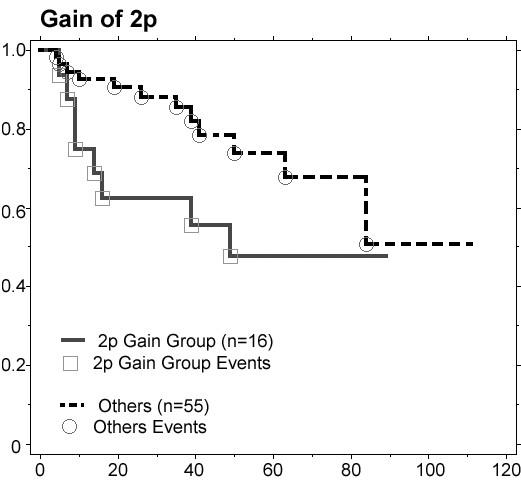
**Survival analysis of patients stratified by gain of the long arm of chromosome 2**. Kaplan-Meier analysis of overall survival (OS) in 71 patients with medulloblastoma stratified by gain of 2p: sixteen patients with 2p gain compared to 55 others without 2p gain. Follow-up is expressed as time from diagnosis in months.

Another common CNA, gain of 2p (minimal region 2p23-p24) detected in 22.5% of tumors (n = 16) displayed a trend towards worse overall survival (*p *= 0.0848) [Table 3, Figure 3]. Amplification at 2p23-p24 seen in 5.6% of tumors (n = 4) was also associated with worse overall survival (*p *= 0.0152). However, gain of 2p without 2p23-p24 amplification in 17% of tumors (n = 12) was not associated with outcome.

**Figure 4:**
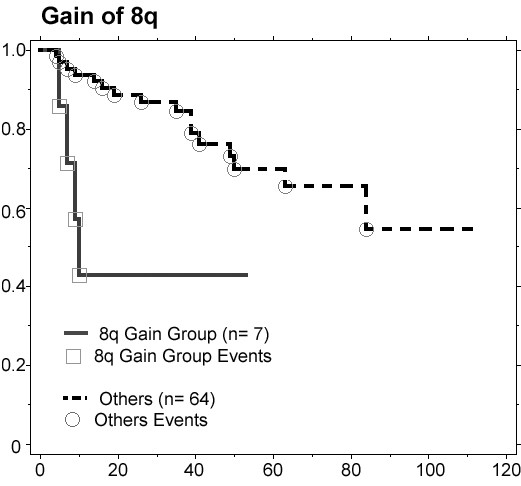
**Survival analysis of patients stratified by gain of the long arm of chromosome 8**. Kaplan-Meier analysis of overall survival (OS) in 71 patients with medulloblastoma stratified by gain of 8q: seven patients with 8q gain and 64 others without 8q gain. Follow-up is expressed as time from diagnosis in months.

Gain of 8q (minimal region 8q23-q24, in 10% of tumors) displayed the strongest association with significantly worse overall and progression-free survival (*p *= 0.0141 and 0.0004, respectively) [Table 3, Figure 4]. The gain of 8q was also associated with adverse outcome in the well described high-risk group of patients were younger than three years of age at diagnosis (n = 14) (*p = *0.0023). One of seven tumors with 8q gain also displayed large cell/anaplastic histology with partial loss of 8p23-q22 and amplification at 8q23-q24, which includes the *MYC *locus (8q24.12-q24.13). When the single case of 8q amplification is excluded from the analysis, the other six patients with gain of 8q22-q24 displayed an even stronger association with overall survival (*p *= 0.0005).

We performed multivariate analysis to test the significance of 8q gain with regard to outcome. When controlled for widely accepted prognostic factors (i.e. extent of resection, age relative to 3 years and metastatic stage at diagnosis), 8q gain is still significantly associated with both PFS and OS (*p *= 0.003 and 0.013, respectively) [see Additional file 1].

### Adverse outcome is not associated with *MYC *or *MYCN *expression

To address the potential contribution of *MYC *or *MYCN*, we screened gene expression microarray data of 27 medulloblastoma specimens that were among 64 previously reported tumors [6]. We did not discern significant upregulation of *MYCN *or other 2p-mapped gene expression in the seven tumors with 2p gain or amplification relative to the twenty tumors without 2p gain [Table 4]. Nor did we detect an association between *MYCN *expression and overall survival in the larger group of 64 medulloblastomas. We corroborated the microarray data with qRT-RTPCR in 12 primary medulloblastomas and determined that *MYCN *was not significantly overexpressed in tumors with gain or amplifications of 2p, compared to others (*p *= 0.31) [Table 4].

We also examined the possible contribution of *MYC *expression to the clinical significance of 8q gain. Higher levels of *MYC *expression correlated with slightly worse outcome among the larger group of 64 medulloblastomas, although failed to achieve statistical significance (in agreement with previous results) [6]. In addition, *MYC *was not consistently overexpressed in microarray-analyzed tumors with 8q gain (n = 3) [Table 4]. We quantitated *MYC *in twelve available tumor specimens with qRT-RTPCR and found that *MYC *was not significantly upregulated in the two tumors with 8q gain (*t *= 0.49). Not surprisingly, the one tumor with 8q23-q24 amplification displayed the highest *MYC *level, while low copy number gains of 8q did not correlate with higher *MYC *levels in either microarray- or qRT-RTPCR-tested specimens. As tested by qRT-RTPCR, relative *MYC *expression correlated highly with microarray results (R^2 ^= 0.99). Overall, these data suggest that *MYC *expression, like 8q amplification, is not entirely responsible for the association of 8q gain with survival.

### Independent of 8q status, adverse outcome is associated with expression of 8q-mapped genes: *EEF1D*, *RPL30*, and *RPS20*

Since *MYC *amplification and expression were not associated with overall survival in our series, we analyzed gene expression data to identify other candidate genes affected by this specific CNA. Of the 27 medulloblastomas with both CGH and microarray data, 11% displayed 8q gain, whose expression profiles were compared to the other 89% of tumors. Of the 149 genes with at least 1.5-fold differential expression levels, 27 genes were upregulated in 8q gain tumors and five genes mapped to 8q. Since gene dosage effects related to chromosomal gains may contribute to higher relative gene expression, we sought to define those candidate genes significantly associated with clinical outcome.

**Figure 5:**
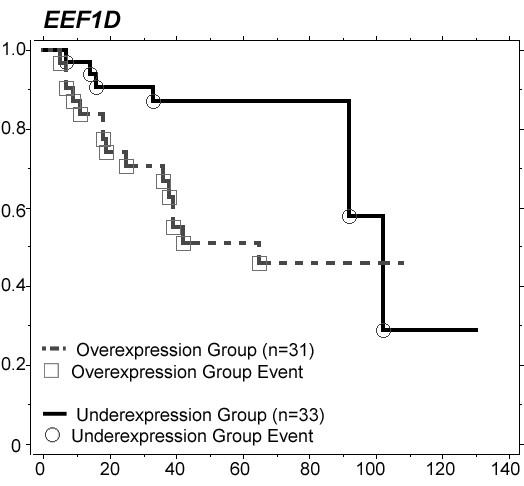
**Survival analysis of patients stratified by expression of eukaryotic translation elongation factor 1 delta (*EEF1D*)**. Kaplan-Meier analysis of overall survival in 64 patients with medulloblastoma based on Hu6800FL array data for the 8q-mapped candidate gene, eukaryotic translation elongation factor 1 delta (*EEF1D*). We calculated the mean between the median expression level of tumors with 8q gain and the median level of those without it. Patients were stratified into two groups based on their tumor expression level relative to that mean. Follow-up is expressed as time from diagnosis in months.

**Figure 6:**
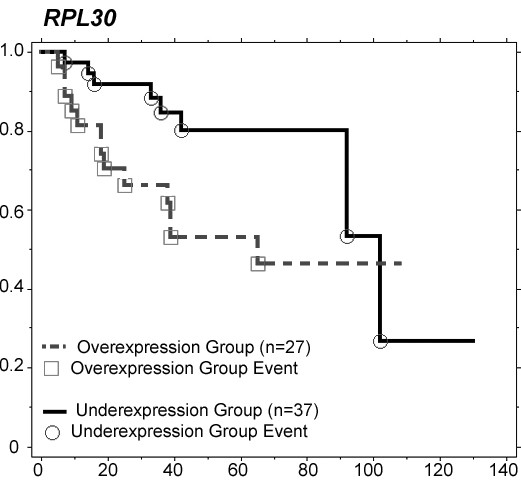
**Survival analysis of patients stratified by expression of ribosomal protein L30 (*RPL30*)**. Kaplan-Meier analysis of overall survival in 64 patients with medulloblastoma based on Hu6800FL array data for the 8q-mapped candidate gene, ribosomal protein L30 (*RPL30*). We calculated the mean between the median expression level of tumors with 8q gain and the median level of those without it. Patients were stratified into two groups based on their tumor expression level relative to that mean. Follow-up is expressed as time from diagnosis in months.

**Figure 7:**
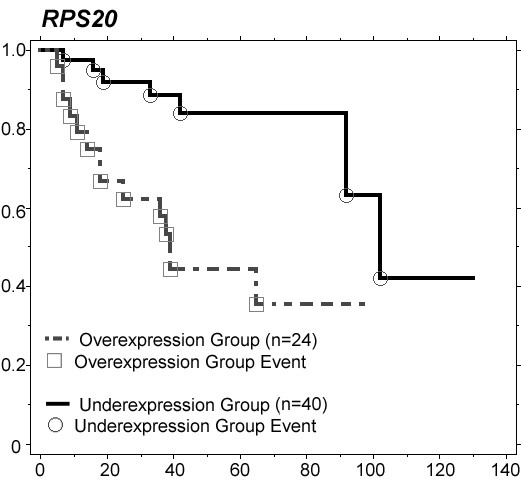
**Survival analysis of patients stratified by expression of ribosomal protein S20 (*RPS20*) expression**. Kaplan-Meier analysis of overall survival in 64 patients with medulloblastoma based on Hu6800FL array data for the 8q-mapped candidate gene, ribosomal protein S20 (*RPS20*). We calculated the mean between the median expression level of tumors with 8q gain and the median level of those without it. Patients were stratified into two groups based on their tumor expression level relative to that mean. Follow-up is expressed as time from diagnosis in months.

In the larger group of 64 microarray-profiled tumors, the expression levels of three of five 8q-mapped genes were adversely associated with overall and progression-free survival: eukaryotic translation elongation factor 1D (*EEF1D;p *values, 0.014 (OS) and 0.0085 (PFS), log-rank), ribosomal protein L30 (*RPL30;p *values, 0.014 (OS) and 0.0018 (PFS)), and ribosomal protein S20 (*RPS20;p *values, 0.0005 (OS) and 0.0213 (PFS)) [Table 4; Figures 5, 6, 7]. In fact, we detected elevated levels of these genes in many tumors without 8q gain. This suggests that not only do these candidate genes contribute to outcome in patients with 8q gain tumors, but also in those regardless of their cytogenetic profiles. The expression levels of *EEF1D*, *RPL30*, and *RPS20 *did not correlate with *MYC *expression in the 64 microarray-profiled tumors.

Because the 64 tumor specimens profiled on Affymetrix HuGeneFL, were unavailable for further analysis by qRT-RTPCR, we could not validate candidate gene expression directly. Alternatively, we assayed the expression of *EEF1D*, *RPL30*, and *RPS20 *with qRT-RTPCR in twelve available medulloblastoma specimens, including those analyzed by Affymetrix U133 Plus 2.0 microarrays [Table 4]. To corroborate the tumor profiles, we compared the expression of each candidate gene by qRT-RTPCR with the new set of microarray-generated data on Affymetrix U133 Plus 2.0. As expected, each candidate gene demonstrated a broader range of differential expression by qRT-RTPCR than by microarray, consistent with signal compression [23]. Our qRT-RTPCR results correlated well with the expression patterns noted in microarray data (R^2^, 0.53–0.90) [Table 4], indicating that observed expression levels detected by microarray analysis likely reflect tumor differences.

## Discussion

We report the results of our CGH analysis of a large series of medulloblastoma and the application of CNA-based outcome analysis of expression datasets to identify candidate genes. The relative frequencies of various CNAs are consistent with previous CGH reports [7,9-12]. The most common aberrations involved chromosome 17, including loss of 17p and gain of 17q, which were not significantly associated with outcome in our series. Several reports have studied the clinical significance of CNAs, in particular involving chromosome 17, but with discordant results. Loss of 17p correlated with survival in several series [24-27]. Mendrzyk *et al *[28] and Pan *et al *[10] have described adverse outcomes associated with combined 17p loss and 17q gain, as in iso17q, respectively. Negative studies include those of Biegel *et al *[29], Emadian *et al *[30], Jung *et al *[31], and Nicholson *et al *[32]. In general, these studies evaluated fewer samples or follow-up was limited. In addition, multiple clinical factors probably contribute to discrepancies among different series.

Our CGH-based outcome analysis reveals a significant association between 8q gain (minimal common region 8q23-q24) and worse overall survival, confirmed by multivariate analysis by controlling for three known prognostic factors. We detected 8q gain in only seven cases of the 71 tumor specimens analyzed. The relatively low frequency of 8q gain probably prevented detection of its clinical significance in other series. *MYC *amplification has been identified as a marker of poor prognosis, but previous studies did not further characterize cytogenetic changes involving 8q. In fact, excluding the only patient with tumor amplification at 8q23-q24 from the outcome analysis actually strengthens the association of 8q gain with overall survival. Although only a minority of specimens was available for confirmatory qRT-RTPCR quantitation, in those tumors tested, *MYC *levels correlated well with microarray results. *MYC *expression levels, however, were not associated with survival in our series, in contrast with previous reports [33]. Overall, our results indicate that biological factors associated with 8q gain other than *MYC *achieve clinical significance in our series of medulloblastoma patients. Due to the relatively low frequency of 8q gain, however, its prognostic significance requires confirmation in studies with larger sample size.

Our CGH and gene expression analysis indicate that other genes mapped to 8q may contribute to clinical outcome. We screened microarray profiles in an overlapping subset of tumors to identify differentially expressed candidate genes overexpressed in specimens with 8q gain. The expression of three candidate genes were associated with overall and progression-free survival: *EEF1D*, *RPL30*, and *RPS20*. By analyzing the larger group of 64 patients, we confirmed the association of these candidate genes with outcome. In fact, among the 64 patients, higher expression of *EEF1D*, *RPL30*, and *RPS20 *correlated with worse overall and progression-free survival regardless of cytogenetic profile. Since we also detected overexpression of these candidate genes in tumor samples without 8q gain, alternate mechanisms of induction may otherwise contribute to tumor phenotype and clinical outcome.

These candidate genes represent a novel group involved in translational regulation and have not been previously associated with outcome in medulloblastoma. Although we previously reported that *RPL30 *expression was associated with classic histology, the smaller expression differences precluded prior detection and association with survival [6]. In that study, we employed a two-class comparison of array profiles based on an unsupervised clustering algorithm (self-organizing map) to define a multi-gene predictor of adverse outcome. The multi-gene predictor included several other ribosomal genes, but did not include *EEF1D*, *RPL30*, or *RPS20*. All three candidate genes are involved in ribosomal functions: *RPS20 *encodes a component of the 60S ribosomal subunit, and *RPL30 *part of the 40S subunit. The *EEF1D *protein contributes to delivery of t-RNA to ribosomes. The overexpression of *EEF1D *has been associated with advanced tumor stage in gastrointestinal carcinomas and *EEF1D *reportedly displays oncogenic properties *in vitro *[34-36]. This supports the hypothesis that increased expression of ribosomal genes confers a growth advantage [37].

Indeed, accumulating evidence indicates that aberrant regulation of ribosomes, their components, and their functions can be linked to cellular transformation. Several oncogenes and tumor suppressor genes, including *MYC *and *MYCN*, regulate the expression of rRNA and ribosomal proteins [37]. Not surprisingly, cancer cells display increased metabolism and protein synthesis, which requires upregulated ribosomal proteins and rRNA [37]. Since ribosomal proteins and translation factors directly regulate protein synthesis, influencing ribosomal biogenesis is one of the possible mechanisms by which cellular growth controls can be disrupted, resulting in increased proliferation. It remains unclear precisely how deregulation of rRNA and ribosomal functions are involved in tumor formation or progression. Nonetheless, our results suggest that better appreciation of the relative contributions of *EEF1D*, *RPL30*, *RPS20*, and their associated regulatory mechanisms will impact our understanding of medulloblastoma biology. The identification of these three candidate genes indicates that specific mechanisms of ribosomal biosynthesis and translational regulation are certainly worthy of future study in medulloblastoma.

## Conclusion

In summary, our CGH survey of 71 medulloblastomas indicates that the gain of 8q is adversely associated with overall and progression-free survival in patients with medulloblastoma. We applied our cytogenetic results to guide the expression analysis of a subset of twenty-seven tumors and subsequent outcome analysis in the larger group of 64 tumors. By exploiting these complementary genomic datasets, we have implicated the expression of three 8q-mapped candidate genes (*EEF1D*, *RPL30*, and *RPS20*) as adversely associated with overall and progression-free survival, independent of cytogenetic profile. Our results indicate the potential for hypothesis-generation by integrating these global approaches, so that more selective methods may now be applied in future studies of their contributions to tumor biology and clinical outcome.

## Abbreviations

CGH, comparative genomic hybridization; CNA, copy number aberration; EEF1D, eukaryotic translation elongation factor 1D; qRT-RTPCR, quantitative real-time reverse transcription polymerase chain reaction; RPL30, ribosomal protein L30; RPS20, ribosomal protein S20.

## Competing interests

The author(s) declare that they have no competing interests.

## Authors' contributions

MDB, XYL, JD, LMS, and PHR performed CGH analysis. MDB, RCC, JD, LMS, SLP, CCL, TM, and JYHK contributed to gene expression analysis using microarrays and qRT-RTPCR. MDB, RCC, AMA, LP, SLP, and JYHK analyzed tumor pathology, clinical characteristics and outcomes. MDB, JD, TM, PHR, and JYHK analyzed and integrated genomic data. All of the authors made significant contributions to data interpretation and drafting of the manuscript.

## Pre-publication history

The pre-publication history for this paper can be accessed here:



## Supplementary Material

Additional File 1 **Multivariate Analysis of 8q Gain for Overall and Progression-Free Survival Controlled for Other Prognostic Variables**. Multivariate analysis of significance was performed for 8q gain with respect to clinical variables that are widely accepted as prognostically significant: age relative to 3 years old and metastatic stage at diagnosis, and the degree of primary resection. These results confirm the prognostic significance of 8q gain for Overall and Progression-Free Survival (p = 0.013 and p = 0.003. respectively). Abbreviations: CGH8q, 8q gain; age_3, age relative to 3 years; resection, degree of resection; M_status, metastatic (Chang) stage; B, regression coefficient of the model; SE, standard error; df, degrees of freedom; Sig, significance (*p *value based on Wald statistics); Exp(B), exponential function of B. Click here for file

## Figures and Tables

**Table 1:** Patient summary

**CLINICAL CHARACTERISTICS**	**71 tumors **analyzed by CGH	**27 tumors **analyzed by both CGH and microarray	**64 tumors **analyzed by microarray
**Median Age in months **(mean; range)	**80 **(89.4; 7–458)	**73 **(93.6; 7–458)	**74 **(99.9; 7–458)
**Median Follow-up in months **(mean; range)	**36 **(39; 4–109)	**39 **(40.2; 7–80)	**39 **(46; 5–130)
**Number of patients < 3 yr old **[*p *value- OS:PFS]	**14 **[0.139:0.187]	**7 **[0.542:0.421]	**11 **[0.406:**0.0005**]*
**STAGE (Chang) – M0: M+ **[*p *value- OS:PFS]	**54 : 17 **[0.027*:0.111]	**20 : 7 **[0.058:0.222]	**43 : 21 **[0.047*:0.012*]
**SEX- male: female **[*p *value- OS:PFS]	**51 : 20 **[0.875:0.532]	**18 : 9 **[0.729:0.839]	**42 : 22 **[0.827:0.957]
**SURGERY- Gross Total Resection: Subtotal Resection **[*p *value- OS:PFS]	**60 : 11 **[0.0701:0.386]	**18 : 9 **[0.028*:0.427]	**40 : 24 **[0.0502:0.926]
**CHEMOTHERAPY- Standard : PBSCT **[*p *value- OS:PFS]	**50:21 **[0.825:0.972]	**27:0 [NA]**	**64:0 [NA]**
**HISTOLOGY**			
***Classic ***[*p *value- OS:PFS]	**30 **[0.714:0.619]	**16 **[0.461:0.996]	**44 **[0.384:0.377]
***Nodular or Desmoplastic ***[*p *value- OS:PFS]	**21 **[0.558:0.731]	**8 **[0.327:0.987]	**15 **[0.46:0.564]
***Large cell or Anaplastic ***[*p *value- OS:PFS]	**17 **[0.844:0.830]	**NA**	**NA**
***Intermediate or Other ***[*p *value- OS:PFS]	**3 **[0.935:0.995]	**3 **[0.769:0.985]	**5 **[0.736:0.532]

Abbreviations: CGH, comparative genomic hybridization; OS, overall survival; PFS, progression-free survival; PBSCT, peripheral blood stem cell transplant; NA, not applicable.

Log-rank *p *values were calculated for OS and PFS.

*Statistically significant *p *values.

**Table 2:** Most Common Chromosomal Copy Number Aberration Among 71 Medulloblastomas.

**Chromosome Loss**	**Number (%)**	***p *value, log-rank [OS: PFS]**
17p loss	36 (50%)*	0.544: 0.816
17p loss and 17q gain (*consistent with isochromosome 17q*)	23 (32%)*	0.704: 0.874
16q loss	29 (40%)	0.707: 0.889
10q loss	25 (35%)	0.356: 0.252
8p loss	24 (34%)	0.581: 0.423
16p loss	22 (31%)	0.744: 0.736
6q loss	16 (22%)	0.741: 0.345
10p loss	16 (22%)	0.325: 0.386
9q loss	16 (22%)	0.999: 0.703
11p loss	15 (21%)	0.201: 0.212
19p loss	13 (18%)	0.332: 0.282
3q loss	13 (18%)	0.899: 0.439
6p loss	13 (18%)	0.506: 0.095
11q loss	13 (18%)	0.330: 0.375
13q loss	10 (14%)	0.966: 0.611
17q loss	10 (14%)	0.918: 0.889
15q loss	8 (11%)	0.064: 0.596
1p loss	8 (11%)	0.061: 0.167
20q loss	8 (11%)	0.992: 0.780
X loss	8 (11%)	0.958: 0.613
3p loss	7 (10%)	0.476: 0.343
21q loss	7 (10%)	0.397: 0.573

**Chromosome Gain**	**Number (%)**	***p *value, log-rank [OS: PFS]**

17q gain	34 (48%)*	0.794: 0.340
17q gain and 17p loss (*consistent with isochromosome 17q*)	23 (32%)*	0.704: 0.874
7q gain	21 (30%)	0.927: 0.366
2p gain	16 (22%)	0.394: 0.526
7p gain	16 (22%)	0.394: 0.195
18q gain	11 (15%)	0.896: 0.842
X gain	10 (14%)	0.786: 0.868
2q gain	10 (14%)	0.166: 0.356
17p gain	9 (13%)	0.545: 0.885
5p gain	9 (13%)	0.863: 0.688
18p gain	9 (13%)	0.963: 0.652
1q gain	8 (11%)	0.242: 0.088
9p gain	8 (11%)	0.913: 0.893
19 gain	8 (11%)	0.480: 0.403
8q gain	7 (10%)	0.0141**: 0.0004**
3q gain	7 (10%)	0.212: 0.464
5q gain	7 (10%)	0.657: 0.790
6q gain	7 (10%)	0.551: 0.990

* In 23 cases, 17q gain was detected with concurrent 17p loss, and was consistent with i17q.

** Statistically significant differences by log-rank testing.

**Table 3:** Copy Number Aberrations associated with overall and progression-free survival.

**Copy Number Aberration**	**n**	**Overall Survival ***p *value (log-rank)	**Progression-Free Survival ***p *value (log-rank)
**2p gain **(including amplifications)	12 (16)	0.5257 (0.0848)	0.3938 (0.0701)
**8q gain **(including amplifications*)	6 (7)	0.0005** (0.0141**)	< 0.0001** (0.0004**)

* Multivariate analysis controlling for clinical variables (i.e. metastatic stage, degree of resection, and age relative to 3 years) revealed statistically significant differences in OS (*p *value, 0.013) and PFS (*p *value, 0.003).

** Statistically significant differences by log-rank testing.

**Table 4:** Genes of interest mapped to 8q and 2p.

**Gene**	**Cytoband**	**Affymetrix probe set**	**Overall Survival **(*p *value^1^)	**Progression- Free Survival **(*p *value^1^)	**Mean Fold Change by Microarray **(ratio of tumors with CNA: tumors without CNA)^2 ^(*p *value)
**Ribosomal protein S20 (*RPS20*)**	8q12	HG1800-HT1823_at	0.0005*	0.0213*	1.58 (0.025)**
**Ribosomal protein L30 (*RPL30*)**	8q22	HG2873-HT3017_at	0.014*	0.0018*	1.78 (0.0162)**
**Euskaryotic translation elongation factor 1 delta (*EEF1D*)**	8q24.3	Z21507_at	0.014*	0.0085*	1.71 (0.0089)**
***MYC***	8q24.12-q24.13	L00058_at and HG3523-HT4899_s_at	0.218–0.378	0.223–0.506	1.35 and 2.99 (0.38 and 0.26)
***MYCN***	2p24.1	M13241_at	0.403	0.661	1.56 (0.31)

^1 ^Kaplan-Meier analysis of 64 patients stratified by microarray-based gene expression levels using Affymetrix HuGeneFL microarrays. Significance determined by log-rank test.

^2 ^Ratio calculated from expression levels in 27 patient samples with and without the specific CNA (8q or 2p gain), using HuGeneFL microarrays. Significance determined by log-rank test.

* Statistically significant differences in overall survival based on microarray expression levels.

** Statistically significant differences in microarray expression levels among tumors analyzed by both CGH and microarray.

## References

[B1] Strother DR, Pollack IF, Fisher PG, Hunter JV, Woo SY, Pomeroy SL, Rorke LB, Pizzo P, Poplack D (2003). Tumors of the Central Nervous System. Principles and Practice of Pediatric Oncology.

[B2] Ellison D (2002). Classifying the medulloblastoma: insights from morphology and molecular genetics. Neuropathol Appl Neurobiol.

[B3] Giangaspero F, Bigner SH, Giordana MT, Kleihues P, Trojanowski JQ (2000). Medulloblastoma. Pathology and genetics: Tumours of the Nervous System World Health Organization Classification of Tumors.

[B4] Fernandez-Teijeiro A, Betensky RA, Sturla LM, Kim JY, Tamayo P, Pomeroy SL (2004). Combining gene expression profiles and clinical parameters for risk stratification in medulloblastomas. J Clin Oncol.

[B5] Gilbertson RJ, Gajjar A (2005). Molecular biology of medulloblastoma: will it ever make a difference to clinical management?. J Neurooncol.

[B6] Pomeroy SL, Tamayo P, Gaasenbeek M, Sturla LM, Angelo M, McLaughlin ME, Kim JY, Goumnerova LC, Black PM, Lau C, Allen JC, Zagzag D, Olson JM, Curran T, Wetmore C, Biegel JA, Poggio T, Mukherjee S, Rifkin R, Califano A, Stolovitzky G, Louis DN, Mesirov JP, Lander ES, Golub TR (2002). Prediction of central nervous system embryonal tumour outcome based on gene expression. Nature.

[B7] Bayani J, Zielenska M, Marrano P, Kwan NY, Taylor MD, Jay V, Rutka JT, Squire JA (2000). Molecular cytogenetic analysis of medulloblastomas and supratentorial primitive neuroectodermal tumors by using conventional banding, comparative genomic hybridization, and spectral karyotyping. J Neurosurg.

[B8] Eberhart CG, Kepner JL, Goldthwaite PT, Kun LE, Duffner PK, Friedman HS, Strother DR, Burger PC (2002). Histopathologic grading of medulloblastomas: a Pediatric Oncology Group study. Cancer.

[B9] Biegel JA (1999). Cytogenetics and molecular genetics of childhood brain tumors. Neuro-oncol.

[B10] Pan E, Pellarin M, Holmes E, Smirnov I, Misra A, Eberhart CG, Burger PC, Biegel JA, Feuerstein BG (2005). Isochromosome 17q is a negative prognostic factor in poor-risk childhood medulloblastoma patients. Clin Cancer Res.

[B11] Gilhuis HJ, Anderl KL, Boerman RH, Jeuken JM, James CD, Raffel C, Scheithauer BW, Jenkins RB (2000). Comparative genomic hybridization of medulloblastomas and clinical relevance: eleven new cases and a review of the literature. Clin Neurol Neurosurg.

[B12] Tong CY, Hui AB, Yin XL, Pang JC, Zhu XL, Poon WS, Ng HK (2004). Detection of oncogene amplifications in medulloblastomas by comparative genomic hybridization and array-based comparative genomic hybridization. J Neurosurg.

[B13] Blaney SM, Boyett J, Friedman H, Gajjar A, Geyer R, Horowtiz M, Hunt D, Kieran M, Kun L, Packer R, Phillips P, Pollack IF, Prados M, Heideman R (2005). Phase I clinical trial of mafosfamide in infants and children aged 3 years or younger with newly diagnosed embryonal tumors: a pediatric brain tumor consortium study (PBTC-001). J Clin Oncol.

[B14] Duffner PK, Horowitz ME, Krischer JP, Friedman HS, Burger PC, Cohen ME, Sanford RA, Mulhern RK, James HE, Freeman CR, Seidel FG, Kun LE (1993). Postoperative chemotherapy and delayed radiation in children less than three years of age with malignant brain tumors. N Engl J Med.

[B15] Packer RJ, Rood BR, Macdonald TJ (2003). Medulloblastoma: present concepts of stratification into risk groups. Pediatr Neurosurg.

[B16] Thomas PR, Deutsch M, Kepner JL, Boyett JM, Krischer J, Aronin P, Albright L, Allen JC, Packer RJ, Linggood R, Mulhern R, Stehbens JA, Langston J, Stanley P, Duffner P, Rorke L, Cherlow J, Friedman HS, Finlay JL, Vietti TJ, Kun LE (2000). Low-stage medulloblastoma: final analysis of trial comparing standard-dose with reduced-dose neuraxis irradiation. J Clin Oncol.

[B17] Strother D, Ashley D, Kellie SJ, Patel A, Jones-Wallace D, Thompson S, Heideman R, Benaim E, Krance R, Bowman L, Gajjar A (2001). Feasibility of four consecutive high-dose chemotherapy cycles with stem-cell rescue for patients with newly diagnosed medulloblastoma or supratentorial primitive neuroectodermal tumor after craniospinal radiotherapy: results of a collaborative study. J Clin Oncol.

[B18] Sambrook J, Russell DW (2001). Molecular Cloning, a Laboratory Manual New York.

[B19] Kim JY, Sutton ME, Lu DJ, Cho TA, Goumnerova LC, Goritchenko L, Kaufman JR, Lam KK, Billet AL, Tarbell NJ, Wu J, Allen JC, Stiles CD, Segal RA, Pomeroy SL (1999). Activation of neurotrophin-3 receptor TrkC induces apoptosis in medulloblastomas. Cancer Res.

[B20] Rao PH (2005). Comparative genomic hybridization for analysis of changes in DNA copy number in multiple myeloma. Methods Mol Med.

[B21] Li C, Wong WH (2001). Model-based analysis of oligonucleotide arrays: expression index computation and outlier detection. Proc Natl Acad Sci USA.

[B22] Pfaffl MW (2001). A new mathematical model for relative quantification in real-time RT-PCR. Nucleic Acids Res.

[B23] Dallas PB, Gottardo NG, Firth MJ, Beesley AH, Hoffmann K, Terry PA, Freitas JR, Boag JM, Cummings AJ, Kees UR (2005). Gene expression levels assessed by oligonucleotide microarray analysis and quantitative real-time RT-PCR – how well do they correlate?. BMC Genomics.

[B24] Batra SK, McLendon RE, Koo JS, Castelino-Prabhu S, Fuchs HE, Krischer JP, Friedman HS, Bigner DD, Bigner SH (1995). Prognostic implications of chromosome 17p deletions in human medulloblastomas. J Neurooncol.

[B25] Cogen PH, McDonald JD (1996). Tumor suppressor genes and medulloblastoma. J Neurooncol.

[B26] Gilbertson R, Wickramasinghe C, Hernan R, Balaji V, Hunt D, Jones-Wallace D, Crolla J, Perry R, Lunec J, Pearson A, Ellison D (2001). Clinical and molecular stratification of disease risk in medulloblastoma. Br J Cancer.

[B27] Lamont JM, McManamy CS, Pearson AD, Clifford SC, Ellison DW (2004). Combined histopathological and molecular cytogenetic stratification of medulloblastoma patients. Clin Cancer Res.

[B28] Mendrzyk F, Radlwimmer B, Joos S, Kokocinski F, Benner A, Stange DE, Neben K, Fiegler H, Carter NP, Reifenberger G, Korshunov A, Lichter P (2005). Genomic and protein expression profiling identifies CDK6 as novel independent prognostic marker in medulloblastoma. J Clin Oncol.

[B29] Biegel JA, Janss AJ, Raffel C, Sutton L, Rorke LB, Harper JM, Phillips PC (1997). Prognostic significance of chromosome 17p deletions in childhood primitive neuroectodermal tumors (medulloblastomas) of the central nervous system. Clin Cancer Res.

[B30] Emadian SM, McDonald JD, Gerken SC, Fults D (1996). Correlation of chromosome 17p loss with clinical outcome in medulloblastoma. Clin Cancer Res.

[B31] Jung HL, Wang KC, Kim SK, Sung KW, Koo HH, Shin HY, Ahn HS, Shin HJ, Cho BK (2004). Loss of heterozygosity analysis of chromosome 17p13.1-13.3 and its correlation with clinical outcome in medulloblastomas. J Neurooncol.

[B32] Nicholson J, Wickramasinghe C, Ross F, Crolla J, Ellison D (2000). Imbalances of chromosome 17 in medulloblastomas determined by comparative genomic hybridisation and fluorescence in situ hybridisation. Mol Pathol.

[B33] Eberhart CG, Kratz J, Wang Y, Summers K, Stearns D, Cohen K, Dang CV, Burger PC (2004). Histopathological and molecular prognostic markers in medulloblastoma: c-myc, N-myc, TrkC, and anaplasia. J Neuropathol Exp Neurol.

[B34] Joseph P, O'Kernick CM, Othumpangat S, Lei YX, Yuan BZ, Ong TM (2004). Expression profile of eukaryotic translation factors in human cancer tissues and cell lines. Mol Carcinog.

[B35] Lei YX, Chen JK, Wu ZL (2002). Blocking the translation elongation factor-1 delta with its antisense mRNA results in a significant reversal of its oncogenic potential. Teratog Carcinog Mutagen.

[B36] Ogawa K, Utsunomiya T, Mimori K, Tanaka Y, Tanaka F, Inoue H, Murayama S, Mori M (2004). Clinical significance of elongation factor-1 delta mRNA expression in oesophageal carcinoma. Br J Cancer.

[B37] Ruggero D, Pandolfi PP (2003). Does the ribosome translate cancer?. Nat Rev Cancer.

