# Supplementary material for: Medulloblastoma outcome is adversely associated with overexpression of EEF1D, RPL30, and RPS20 on the long arm of chromosome 8
Source: BMC Cancer. 2006 Sep 12;6:223. doi: 10.1186/1471-2407-6-223 (PMC1578584; doi:10.1186/1471-2407-6-223)
Supplement: Additional File 1 — Multivariate Analysis of 8q Gain for Overall and Progression-Free Survival Controlled for Other Prognostic Variables. Multivariate analysis of significance was performed for 8q gain with respect to clinical variables that are widely accepted as prognostically significant: age relative to 3 years old and metastatic stage at diagnosis, and the degree of primary resection. These results confirm the prognostic significance of 8q gain for Overall and Progression-Free Survival (p = 0.013 and p = 0.003. respectively). Abbreviations: CGH8q, 8q gain; age_3, age relative to 3 years; resection, degree of resection; M_status, metastatic (Chang) stage; B, regression coefficient of the model; SE, standard error; df, degrees of freedom; Sig, significance (p value based on Wald statistics); Exp(B), exponential function of B. [file 1471-2407-6-223-S1.pdf]

Variables in the Equation

Progression-Free Survival

	B	SE	Wald	df	Sig.	Exp(B)
CGH8q	1.637	.553	8.767	1	.003	5.138
age_3	1.101	.473	5.426	1	.020	3.006
resection	.870	.582	2.240	1	.134	2.388
M_status	.292	.444	.434	1	.510	1.339

Variables in the Equation

Overall Survival

	B	SE	Wald	df	Sig.	Exp(B)
CGH8q	1.531	.615	6.205	1	.013	4.624
age_3	.981	.541	3.286	1	.070	2.667
resection	1.315	.583	5.083	1	.024	3.725
M_status	.341	.466	.535	1	.464	1.406
